# New Insights for Polyphenolic Compounds as Naturally Inspired Proteasome Inhibitors

**DOI:** 10.3390/ph16121712

**Published:** 2023-12-11

**Authors:** Emanuela Marchese, Maria Eugenia Gallo Cantafio, Francesca Alessandra Ambrosio, Roberta Torcasio, Ilenia Valentino, Francesco Trapasso, Giuseppe Viglietto, Stefano Alcaro, Giosuè Costa, Nicola Amodio

**Affiliations:** 1 Dipartimento di Scienze della Salute, Università “Magna Græcia” di Catanzaro, Campus “S. Venuta”, 88100 Catanzaro, Italy; e.marchese@unicz.it (E.M.); alcaro@unicz.it (S.A.); gcosta@unicz.it (G.C.); 2 Dipartimento di Medicina Sperimentale e Clinica, Università degli Studi “Magna Græcia” di Catanzaro, Campus “S. Venuta”, Viale Europa, 88100 Catanzaro, Italy; mariaeugenia.gallocantafio@unicz.it (M.E.G.C.); robertatorcasio1@gmail.com (R.T.); ilenia.valentino30@gmail.com (I.V.); trapasso@unicz.it (F.T.); viglietto@unicz.it (G.V.); 3 Net4Science Academic Spin-Off, Università “Magna Græcia” di Catanzaro, Campus “S. Venuta”, 88100 Catanzaro, Italy; 4 Associazione CRISEA—Centro di Ricerca e Servizi Avanzati per l’Innovazione Rurale, Loc. Condoleo, 88055 Belcastro, Italy

**Keywords:** natural metabolites, polyphenols, proteasome inhibitors, multiple myeloma, virtual screening studies, DrugBank

## Abstract

Polyphenols, an important class of natural products, are widely distributed in plant-based foods. These compounds are endowed with several biological activities and exert protective effects in various physiopathological contexts, including cancer. We herein investigated novel potential mechanisms of action of polyphenols, focusing on the proteasome, which has emerged as an attractive therapeutic target in cancers such as multiple myeloma. We carried out a structure-based virtual screening study using the DrugBank database as a repository of FDA-approved polyphenolic molecules. Starting from 86 polyphenolic compounds, based on the theoretical binding affinity and the interactions established with key residues of the chymotrypsin binding site, we selected 2 promising candidates, namely Hesperidin and Diosmin. The further assessment of the biologic activity highlighted, for the first time, the capability of these two molecules to inhibit the β5-proteasome activity and to exert anti-tumor activity against proteasome inhibitor-sensitive or resistant multiple myeloma cell lines.

## 1. Introduction

Polyphenolic compounds (PCs) comprise a significant part of natural secondary metabolites, widely distributed in plant-based foods [1,2]. According to the number of phenol rings and the structural elements that bind these moieties to each other, PCs can be divided into many classes, mainly represented by flavonoids, phenolic acids, tannins, stilbenes, and lignans [3,4]. In addition, a broad variety of sugar substitutes can be found in polyphenols, typically containing only one unit, and occasionally more than one [5]. The physical properties of PCs, resulting directly from their chemical structure, contribute to the sensory and nutritional characteristics of plants and foods in which they are contained [6]. In particular, the presence of multiple hydroxyl groups in aromatic systems accounts for the capacity to interact and quench reactive oxygen species (ROS) [7]. By counteracting radical propagation in biological systems, polyphenols play an important role in redox balance and the protection of cells and tissues from oxidative stress, thus preventing oxidative damage-related diseases [8,9].

Although antioxidant activity is one of the most recognized functions of polyphenols and their metabolites [10,11,12], they have been also found to hamper the development of many chronic conditions, such as cardiovascular diseases [13], diabetes [14], obesity [15], infections [16], asthma [17], neurodegenerative disorders [18], and cancer [19,20,21]. In the latter case, this natural class of compounds has been shown to impair cell proliferation, tumor growth, angiogenesis, inflammation, and to activate apoptosis [22].

Based on the anti-tumor potential of polyphenols and the multiple biological functions [23], we investigated whether they could also interfere with the proteasome activity. Indeed, the proteasome, due to its role in the degradation of oncogenic proteins and regulation of key cellular pathways, has emerged as an attractive therapeutic target for the development of new anti-cancer drugs [24]. The proteasome functions as the core component of the ubiquitin–proteasome system (UPS) which selectively targets proteins for degradation by attaching ubiquitin molecules to them. Structurally, the proteasome is a large (1500–2000 kDa) multi-subunit protein complex, consisting of a cylindrical core particle, the 20S core particle (CP), and one or two regulatory particles (RP) at one or both ends. In detail, the CP comprises four stacked rings, each containing seven individual subunits. The two outer rings consist of α subunits, which provide structural support, while the two inner rings are formed by β subunits, with proteolytic activity, which are further classified into three types: β1, β2, and β5. The RP, on the other hand, are involved in recognizing and unfolding ubiquitinated proteins, facilitating their entry into the CP for degradation [25]. The overall structure of this unique multi-catalytic enzyme enables the efficient recognition, degradation, and recycling of cellular proteins, playing a key role in maintaining cellular homeostasis. Dysregulation of the proteasome has been implicated in the pathogenesis of several malignancies, including cancer.

The development of proteasome inhibitors (PIs) has revolutionized cancer treatment, particularly in hematological malignancies as multiple myeloma (MM) [26]. MM is characterized by an abnormal plasma cell proliferation and accumulation in the bone marrow that displaces healthy blood cells. Additionally, these malignant plasma cells can generate abnormal antibodies known as monoclonal proteins, leading to various complications [27]. The introduction of PIs, such as bortezomib (Velcade^®^) [28], carfilzomib (Kyprolis^®^) [29], and ixazomib (Ninlaro^®^) [30] has significantly improved the treatment outcome for MM patients. These inhibitors have been shown to induce apoptosis, inhibit tumor growth, and overcome drug resistance in MM cells. However, the emergence of drug resistance poses a constant challenge, necessitating the development of new drugs, particularly in the relapsed and refractory setting [31,32,33]. The search for novel compounds with proteasome-inhibiting properties has expanded towards natural products. In this regard, the attention to natural PIs has begun with the successful discovery of marizomib (salinosporamide A) [34], a marine natural irreversible PI extracted from the marine actinomycete *Salinospora tropica*. Recently, we summarized the evidence on natural products capable of inhibiting the proteasome [35], opening a promising avenue for the development of new anti-MM drugs.

Thus, considering PCs as starting molecules for hit identification, in the present work, we carried out a structure-based virtual screening (SBVS) study to identify new potential naturally-inspired proteasome inhibitors [36]. To this aim, the DrugBank [37] database was chosen as a useful repository of polyphenolic molecules already approved by the United States Food and Drug Administration (FDA). Starting from 86 PCs, we selected 2 promising candidates which were then submitted to biological evaluations, confirming their ability to inhibit the chymotrypsin proteasome activity and to exert anti-tumor activity against MM cell lines.

## 2. Results

### 2.1. Molecular Docking Studies

In the effort to identify natural PIs, the DrugBank database, which contains a comprehensive collection of various PCs, was utilized. In detail, the ligand library encompasses a range of FDA-approved polyphenols, including anthocyanins, chalcones, flavanols, flavanones, flavones, and flavonols. To select the molecules of interest, we initially filtered the pool of approved drugs (4336 compounds) based on the presence of a polyphenolic scaffold, resulting in about 80 PCs.

The subset of polyphenols was then screened against the proteasome chymotrypsin-like site (β5 subunit) using a structure-based virtual screening (SBVS) approach. The molecular recognition results were ranked according to the Glide docking score (D-score) value, leading to the selection of 5 compounds (Table 1). Finally, considering the highest theoretical binding affinity as well as the commercial availability, we focused on 2 compounds, namely Diosmin, a flavone glycoside, and Hesperidin, a flavanone glycoside.

**Figure 1:**
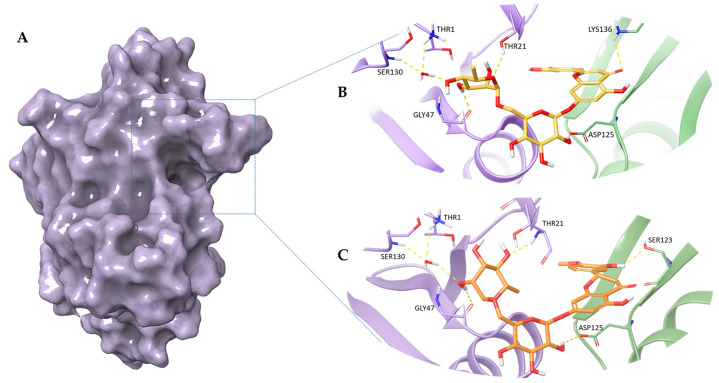
3D representation of (**A**) proteasome chymotripsin-like site (β5 subunit); the best pose of (**B**) Diosmin and (**C**) Hesperidin docked into the proteasome chymotrypsin-like pocket. The Diosmin and Hesperidin are represented, respectively, as yellow and orange carbon sticks. In panels (**B**) and (**C**) the β5 subunit is shown as a violet cartoon, the adjacent β1 subunit is depicted as a light green cartoon, and the residues involved in crucial contacts are reported as violet and light green carbon sticks. The water molecule is shown as red carbon sticks.

By analysing the binding modes of the best pose of the two selected flavonoids, many productive interactions with the proteasome binding site were observed (Figure 1). In detail, Diosmin establishes hydrogen bond interactions with Thr21, Gly47, Lys136, and Asp125 and two water bridges with Thr1 and Ser130 residues. Furthermore, the compound is stabilized by several hydrophobic contacts with Ala20, Ala22, Ala27, Gly47, Val127, and Gln131 residues. Regarding Hesperidin, it is engaged in hydrogen bond interactions with Thr21, Gly47, Ser123, and Asp125, and two water bridges with Thr1 and Ser130. Moreover, the compound is stabilized by several hydrophobic contacts with Arg19, Ala20, Ala22, Val31, Gly47, Gly48, Ala 50, and Gln131 residues.

Overall, molecular docking findings indicate that the two polyphenols are well accommodated within the proteasome chymotrypsin-like pocket, interacting with key aminoacidic residues of the proteasome pocket.

### 2.2. Molecular Dynamics Studies

In order to evaluate the single contributions of hydrogen bonds, hydrophobic, ionic, and water bridge interactions during the molecular dynamics simulations (MDs), we submitted the best docking poses of Diosmin and Hesperidin to 100 ns of MDs (Figure S1). Specifically, Diosmin is able to maintain the pivotal hydrogen bonds and salt bridge interactions with some important residues of the binding site such as Thr1, Thr21, Gly48, and Asp125 during the MDs, as well as hydrophobic interactions with Ala22, Val31 and Met45, promoting the complex’s stability. Concerning Hesperidin, we noticed that it is able to maintain different hydrogen bonds with Tyr107 and Ser129 and water bridges with Asp125 and Gly139 throughout the whole simulation.

**Figure 2:**
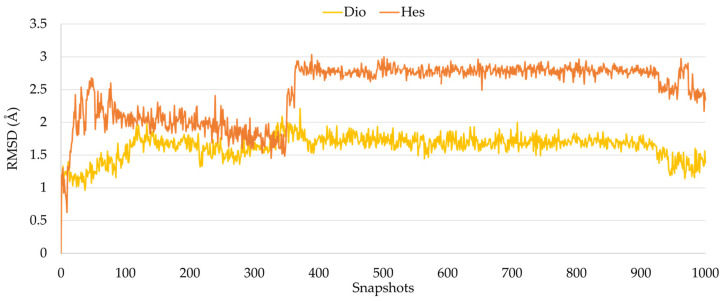
RMSD plot of Diosmin (yellow line) and Hesperidin (orange line) in complex with proteasome. The RMSD values are expressed in Angstrom (Å).

During the whole trajectory, for each ligand, the RMSD trend was calculated, and as reported in Figure 2, Diosmin and Hesperidin were found stable in the protein binding site.

Furthermore, to evaluate the conformational flexibility of the two polyphenols during the whole simulation, the root mean square fluctuation (RMSF), which provide information on the ligand atom position changes throughout the simulation, was calculated.

**Figure 3:**
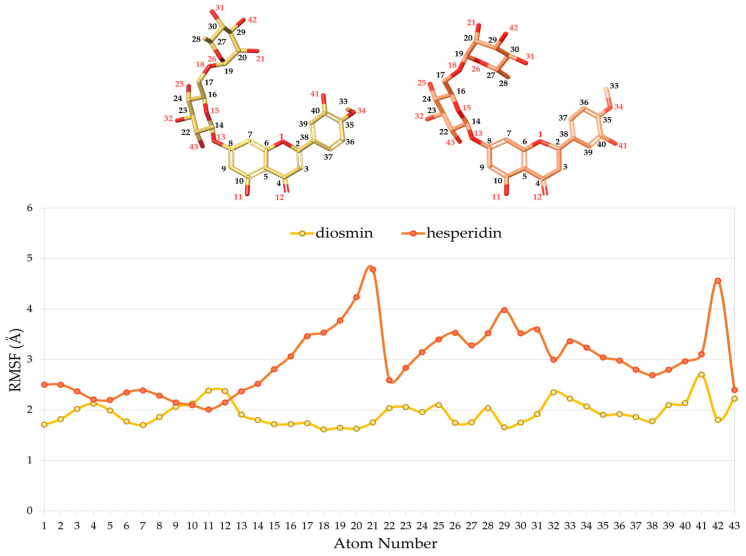
RMSF plot of Diosmin (yellow line) and Hesperidin (orange line). The RMSF values expressed in Angstrom (Å) and the ligand atom number are reported on the ordinate and abscissa axes, respectively.

As reported in Figure 3, the fluctuation of Diosmin within the complex was found to be lower than Hesperidin, due to the presence of a double bond at C2 position which determines the planarity of the aglycon system. Indeed, the RMSF of the methoxyphenol ring linked to C2 assumes values of approximately 2.0 Å and 3.0 Å, in Diosmin and Hesperidin respectively. In addition, the glycosidic portion is also more flexible in Hesperidin than in Diosmin.

Finally, to assess the thermodynamic profile of the two PCs, 1000 snapshots from 100 ns of MD were extracted to calculate the free energy of binding (ΔG-bind). Diosmin and Hesperidin were both associated with a favorable ΔG-bind trend (Figure S2), with average values of −60.53 kcal/mol and −66.14 kcal/mol, respectively, during the entire trajectories.

### 2.3. Hesperidin and Diosmin Inhibit β5 Proteasome Activity In Vitro and in Cell-Based Assays

**Figure 4:**
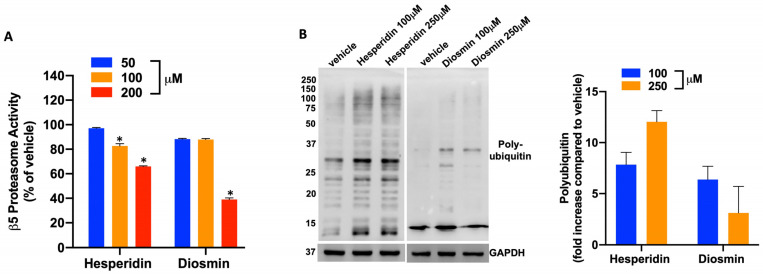
Hesperidin and Diosmin affect proteasome activity. (**A**) Chymotrypsin-like activity evaluated by Proteasome assay (Promega) in a purified enzyme-based format. The luminogenic substrate Suc-LLVY-aminoluciferin, for chymotrypsin-like activity measurement, was added to a buffer system containing proteasome 20S enzyme along with Hesperidin or Diosmin, or vehicle (DMSO) as control. Luminescence was measured after 1 h of incubation time by using GloMax-multi detection system (Promega). (* *p* < 0.05) (**B**) Western Blotting analysis of poly-ubiquitinated proteins in AMO wt cells after Hesperidin or Diosmin treatment at different concentrations (50–100–200 µM) or DMSO as control. Histogram bars represent the densitometric analysis of poly-ubiquitin fold increase after Hesperidin or Diosmin treatment compared to DMSO. GAPDH was used as loading control.

To confirm the above-reported in silico findings, we carried out an in vitro assay using purified β5 proteasome subunit. Incubation of the catalytically active recombinant β5 proteasome subunit with Hesperidin or Diosmin resulted in the inhibition of the β5 enzymatic activity, which was particularly evident at 200 μM concentration (Figure 4A). We next evaluated whether Hesperidin- or Diosmin-treated MM cells could accumulate poly-ubiquitinated proteins as a result of the proteasome inhibition. WB analysis of AMO-1 cell lysates, 48 h after treatment with Hesperidin or Diosmin, evidenced a significant upregulation of poly-ubiquitin species after treatment with both compounds (Figure 4B), thus confirming, in a cell-based assay, the results obtained in vitro.

### 2.4. Hesperidin and Diosmin Exert Anti-Tumor Activity against PI-Sensitive and PI-Resistant MM Cells

**Figure 5:**
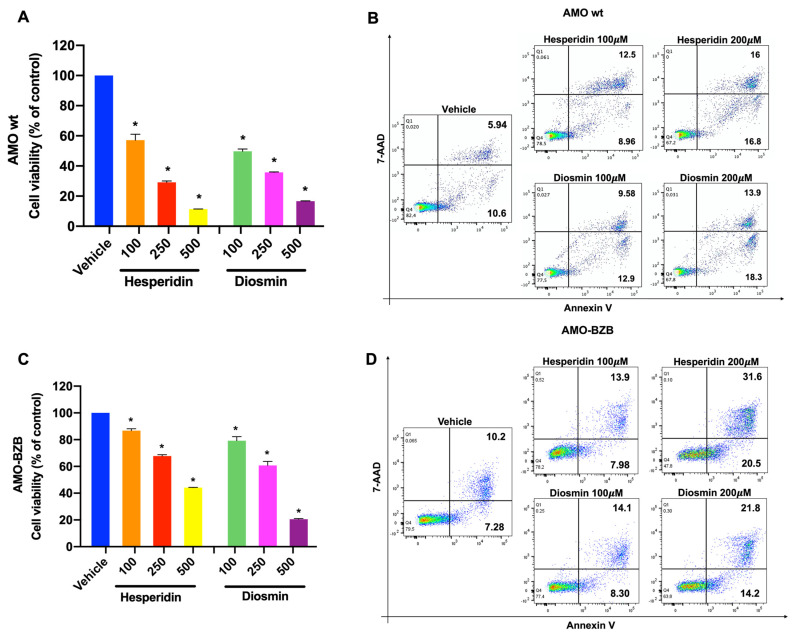
In vitro anti-MM activity of Hesperidin and Diosmin. Cell viability, measured by CTG assay, in AMO wt (**A**) and AMO-BZB (**C**) cells, after 48 h exposure to Hesperidin or Diosmin at different concentrations (100–250–500 µM), or vehicle (DMSO) as control. Data are represented as percentage of Hes-treated and Dios-treated cells compared to DMSO-treated cells. (* *p* < 0.01 compared to DSMO). Flow cytometric analysis of Annexin V/7-AAD stained AMO wt (**B**) and AMO-BZB (**D**) cells 48 h after treatment with different concentrations of Hesperidin or Diosmin (100–250–500 µM). Dot plots are representative of an independent biological replicate (*n* = 3).

We finally tested whether Diosmin or Hesperidin-dependent inhibition of the β5 proteasome activity could in turn affect the viability of MM cells. AMO-1 cells were treated with increasing concentrations of Hesperidin or Diosmin, and then cell viability was assessed using the CTG assay (Figure 5A,C and Figure S3). Notably, Hesperidin or Diosmin treatment reduced MM viability in a dose-dependent manner, and this effect was accompanied by an increase in Annexin-V positive cells, demonstrating apoptosis induction by both compounds.

Finally, to evaluate whether Hesperidin or Diosmin could be active also in bortezomib-resistant cells, PI-resistant AMO-BZB cells were treated with Hesperidin or Diosmin and next analyzed for cell viability and apoptosis. Interestingly, both Hesperidin and Diosmin were able to decrease AMO-BZB cell viability, triggering apoptosis at an extent comparable to AMO-1 cells (Figure 5B,D), suggesting that these natural PIs could be likely effective in PI-resistant settings.

## 3. Discussion

Diosmin (diosmetin 7-O-rutinoside) and Hesperidin (hesperitin 7-O-rutinoside) are two notable natural metabolites, commonly found in citrus fruits belonging to the rutaceae family, such as tangerine (*Citrus reticulata*) [38]. Structurally, they exhibit significant similarities, however, a differentiating feature of Diosmin is the presence of a double bond between two carbon atoms in the C-ring. This structural distinction arises from the natural oxidation process of Hesperidin, a flavanone glycoside, to Diosmin, a flavone glycoside.

So far, numerous in vitro and in vivo studies have uncovered well-established positive effects of both natural metabolites, including antioxidant, anti-inflammatory, antimicrobial, antidiabetic, and anticancer properties [39].

In this study, by means of in silico and experimental studies, we highlighted the capability of Diosmin and Hesperidin to bind and inhibit the β5 proteasome activity, and to trigger anti-tumor activity against multiple myeloma cells.

In detail, our recognition results revealed that the two PCs are well accommodated within the β5 subunit of the proteasome. Examining the best docking poses, we could notice interactions with pivotal aminoacidic residues of the binding pocket in agreement with data already reported in literature [40,41,42,43]. Specifically, our compounds were able to mimic the behavior of ixazomib. In fact, as already shown for this drug belonging to the third-generation proteasome inhibitors, we observed interactions with Thr1, Arg19, Ala20, Thr21, Ala22, Gly47, Gly48, Ala50, Asp125 and Ser130. Moreover, our molecular dynamics simulation results confirmed the stability of the proteasome chymotrypsin subunit in the presence of both compounds.

In silico predictions were further corroborated by in vitro and cell-based assays, underscoring the ability of the two PCs to inhibit the β5 proteasome activity, resulting in a significant upregulation of poly-ubiquitin species.

We finally investigated whether the inhibition of the β5 proteasome activity could in turn impair the viability of MM cells and demonstrate that Diosmin and Hesperidin could reduce viability in a dose-dependent manner, leading to an increase in Annexin-V positive cells, suggestive of apoptosis induction. Notably, both Diosmin and Hesperidin exhibited anti-MM activity in bortezomib-resistant cells, reducing AMO-BZB cell viability and triggering apoptosis at an extent comparable to parental cells, suggesting that these natural PIs could potentially be effective in PI-resistant settings.

In summary, our results highlight the ability of two well-known flavonoids, approved for the treatment of chronic venous insufficiency (CVI) [44,45,46,47], to inhibit the proteasome activity. Therefore, our data strengthens the evidence that polyphenols are a class of natural compounds able to inhibit the proteasome activity [48,49,50].

## 4. Materials and Methods

### 4.1. Computational Studies

Molecular modeling analysis was carried out starting from the crystal structure of the human 20S proteasome complex with Ixazomib, deposited in the Protein Data Bank (PDB) with the PDB code 5LF7 [40]. The receptor structure was prepared with Protein Preparation Wizard [51] tool of Maestro suite (Schrödinger Release 2018-1), using the OLPS_2005 force field [52]. Crystallographic buffer components were removed, missing side chains were constructed using the Prime module, hydrogen atoms were added, and the protonation states of the side chains were assigned to pH 7.4 [53]. To assess the reliability of our molecular recognition approach, redocking calculations were performed using the Glide Standard Protocol (SP) algorithm [54], as already reported in our previously published works [41,42].

For the virtual screening studies, we used a library of 80 PCs obtained from the DrugBank database [37]. The library was prepared using the LigPrep tool [55] of Maestro suite (Schrödinger Release 2018-1), hydrogens were added, salts were removed, and ionization states were calculated at pH 7.4. Each structure was submitted to 10,000 MacroModel [56] minimization steps using the OPLS_2005 as force field.

Docking studies were performed using Glide v. 7.8 [54] standard precision (SP) algorithm, generating 25 poses for each ligand. Considering the theoretical binding affinity score value of the best hits, we selected all the structures with a docking score value within 1 kcal/mol from the best hit.

Finally, considering their higher theoretical binding affinity and verifying their commercial availability in several vendor databases, the 2 compounds Diosmin and Hesperidin were chosen.

The best docking pose of the two selected compounds was submitted to 100 ns of molecular dynamics simulations (MDs) run using the Desmond package v. 5.3. [57]. The system was immersed in an orthorhombic box of TIP4P water molecules and counter ions were added to neutralize the system charge. The system temperature was set at 300 K and the NPT ensemble was selected.

One thousand snapshots from 100 ns of MDs were subjected to molecular mechanism generalized Born surface area (MM-GBSA) calculations [53,58], using VSGB as the solvation model and OPLS_2005 as the force field.

### 4.2. MM Cell Lines

The human MM cell lines AMO-1 and the PI-resistant AMO-BZB, were kindly provided by Dr. C. Driessen (University of Tubingen, Tubingen, Germany), and cultured in RPMI-1640 medium (Corning, Corning, NY, USA), containing 100 U/mL penicillin, 100mg/mL streptomycin (Gibco, Life Technologies, Carlsbad, CA, USA), 2 µmol/L glutamine (Gibco, Life Technologies), supplemented with 10% heat inactivated FBS (Gibco, Life Technologies), and incubated at 37 °C in a 5% CO_2_ atmosphere, as previously reported [21]. AMO-BZB cells were obtained after exposure to increased concentrations of bortezomib, and finally cultured in FBS-supplemented medium containing 20 nM bortezomib. Cells were periodically tested to rule out mycoplasma contamination using the MycoAlert Mycoplasma Detection kit (Lonza, Basel, Switzerland).

### 4.3. Cell Viability Assay

To evaluate the percentage of viable MM cells after Hesperidin or Diosmin treatment, we used Cell Titer Glo (CTG) assay (Promega, Madison, WI, USA), based on quantification of the ATP present in cell lysate, as indicator of metabolically active cells, according to manufacturer’s instructions. Briefly, MM cells were seeded in opaque 96 well plates in triplicate and treated with different concentrations of Hesperidin and Diosmin. After 24, 48 or 72 h, the single reagent was added directly to cell cultured in a serum-supplemented medium and incubated for 60 min at room temperature. Luminescence was measured by using GloMax-multi detection system (Promega).

Data are represented as the average of three independent experiments performed in triplicate.

### 4.4. Apoptosis Assay

Apoptosis and cell viability were evaluated using Annexin V/7-Amminoactinomycin (7-AAD) flow cytometry assay, according to PE Annexin V Apoptosis detection kit (Thermo Fisher Scientific, Waltham, MA, USA) protocol, as reported [59]. Briefly, after treatment with different doses of Hesperidin and Diosmin, AMO-1 and AMO-BZB cells were harvested and washed in PBS 1x, then stained in a 5 mL polystyrene tubes by using Annexin V PE and 7-AAD probes, according to manufacturer’s instruction (Thermo Fisher Scientific), and next incubated at room temperature for 15 min. The sample analysis was performed using FACS Fortessa X-20 (BD Bioscience, Franklin Lakes, NJ, USA). Results were analyzed by using FlowJo software version 10, and reported as histogram bars representing the percentage of total Annexin V positive cells.

The assay was performed in triplicate and represented as means ± SD for each condition.

### 4.5. Western Blotting (WB)

SDS-PAGE and WB were performed according to standard protocols. Total cell proteins were extracted using NP40 lysis buffer supplemented with Halt protease and phosphatase inhibitor cocktail (Thermo Fisher Scientific). Whole cell lysate was separated using SDS-Acrylamide gels (Bio-Rad, Hercules, CA, USA), and electro-transferred on 0.45 µm nitrocellulose membranes (Bio-Rad, USA) using the transblot system (Bio-Rad, USA). The membranes were immunoblotted overnight at 4 °C with each of the following antibodies: ubiquitin (#3936) and GAPDH (#5174), purchased from Cell Signaling Technology (Danvers, MA, USA).

### 4.6. In Vitro Proteasome Assay

Proteasome activity was measured by Proteasome-Glo Assay (Promega), according to manufacturer’s instructions. To evaluate the specific β5 chymotrypsin-like caspase activity, the luminogenic substrate containing the Suc-LLVY-aminoluciferin for chymotrypsin-like caspase subunit was added to a buffer system in a purified enzyme-based format, optimized for the measurement of the specific proteasome activity and luciferase activity. The assay reagents were added to the test chemical samples, containing different concentrations of Hesperidin or Diosmin along with the human purified proteasome 20S enzyme (1 µg/mL) (Enzo Life Sciences, Farmingdale, NY, USA) that cleaves the substrates, releasing luciferin, next consumed by luciferase, producing luminescence corresponding to the enzyme activity or inhibition. Luminescence was measured in a white 96 well plate by using GloMax-multi detection system (Promega).

### 4.7. Statistical Analysis

Each experiment was carried out at least three times and values are reported as mean ± s.d. Student’s *t*-test was used for the comparisons between groups, while statistical significance of differences among multiple groups was determined by GraphPad Prism software (www.graphpad.com). Graphs were obtained using GraphPad Prism version 8.0 (GraphPad Software, La Jolla, CA, USA), where *p*-value < 0.05 were accepted as statistically significant.

## 5. Conclusions

Polyphenols represent an important class of natural compounds biosynthesized by plants that, due to the presence of various phenolic groups, counteract oxidative stress in several physiopathological conditions. Herein, looking beyond the well-recognized antioxidant properties, we explored novel potential modes of action of these molecules which can likely contribute to explain their anti-tumor activity.

Strikingly, we demonstrate that Diosmin and Hesperidin can act as chemical entities able to bind and inhibit specific macromolecular targets such as the proteasome.

In detail, by combining in silico and in vitro studies, Diosmin and Hesperidin were identified as new potential natural proteasome inhibitors through their ability to inhibit the chymotrypsin-like proteasome activity. Our results underscore the potential of these polyphenolic compounds to be novel anti-MM agents, potentially effective even after the emergence of resistance to conventional PIs.

Based on these findings, we will design structural modifications of the two identified natural metabolites to enhance their potency, and we will also test the β5-inhibitory activity of the other hits emerged from our screening. Finally, we plan to combine Diosmin and Hesperidin in order to evaluate possible synergistic effects on proteasome inhibition.

## Figures and Tables

**Table 1:** Name, DrugBank ID, 2D structure, and docking score of the best selected polyphenolic compounds.

Name	DrugBank ID	2D Structure	D-Score *
Diosmin	DB08995	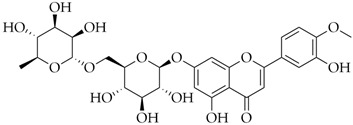	−8.26
Hesperidin	DB04703	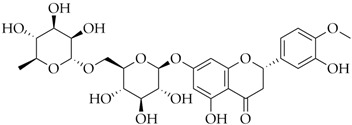	−8.14
Polydatin	DB11263	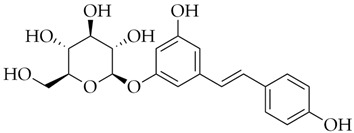	−7.99
Cromoglicic Acid	DB01003	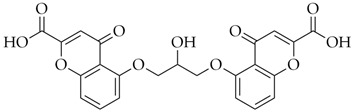	−7.98
Curcumin	DB11672	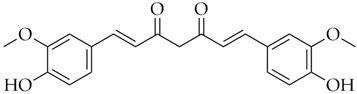	−7.79

* docking score values are expressed in kcal/mol.

## Data Availability

Data is contained within the article and supplementary material.

## Supplementary Materials

The following supporting information can be downloaded at: https://www.mdpi.com/article/10.3390/ph16121712/s1, Figure S1: Protein-ligand interactions of (a) Diosmin and (b) Hesperidin monitored during molecular dynamics simulations. Hydrogen bonds, hydrophobic, ionic and water bridges contacts are represented in green, violet, red and blue, respectively; Figure S2: Plots of MM/GBSA trend for Diosmin (blue line) and Hesperidin (salmon line) in complex to the proteasome chymotrypsin-like site, during 100 ns of MDs; Figure S3: IC50 values of Hesperidin and Diosmin in MM cell lines. Cells were treated with different concentrations of the two PCs for 48 h, and cell viability assayed using the CTG method. IC50 values were calculated using GraphPad Prism 8 software and reported as mean of three independent experiments ± SD.
