# Supplementary material for: New Insights for Polyphenolic Compounds as Naturally Inspired Proteasome Inhibitors
Source: Pharmaceuticals (Basel). 2023 Dec 11;16(12):1712. doi: 10.3390/ph16121712 (PMC10747119; doi:10.3390/ph16121712)
Supplement: Supplementary file 1 [file pharmaceuticals-16-01712-s001.zip › pharmaceuticals-2708831-SI.pdf]

Supporting Information

New Insights for Polyphenolic Compounds as Naturally Inspired Proteasome Inhibitors

Emanuela Marchese [1,†], Maria Eugenia Gallo Cantafio [2,†], Francesca Alessandra Ambrosio [2,*], Roberta Torcasio [2], Ilenia Valentino [2], Francesco Trapasso [2], Giuseppe Viglietto [2], Stefano Alcaro [1,3,4], Giosuè Costa [1,3,‡] and Nicola Amodio [2,*,‡]

[1] Dipartimento di Scienze della Salute, Università "Magna Græcia" di Catanzaro, Campus "S. Venuta", 88100 Catanzaro, Italy; e.marchese@unicz.it (E.M.); alcaro@unicz.it (S.A.); gcosta@unicz.it (G.C.)

[2] Dipartimento di Medicina Sperimentale e Clinica, Università degli Studi "Magna Græcia" di Catanzaro, Campus "S. Venuta", Viale Europa, 88100 Catanzaro, Italy; mariaeugenia.gallocantafio@unicz.it (M.E.G.C.); robertatorcasio1@gmail.com (R.T.); ilenia.valentino30@gmail.com (I.V.); trapasso@unicz.it (F.T.); viglietto@unicz.it (G.V.)

[3] Net4Science Academic Spin-Off, Università "Magna Græcia" di Catanzaro, Campus "S. Venuta", 88100 Catanzaro, Italy

[4] Associazione CRISEA—Centro di Ricerca e Servizi Avanzati per l'Innovazione Rurale, Loc. Condoleo, 88055 Belcastro, Italy

[*] Correspondence: ambrosio@unicz.it (F.A.A.); amodio@unicz.it (N.A.)

[†] These authors contributed equally to this work.

[‡] These authors are co-last authors.

Table of content

Figure S1. Protein-ligand interactions of **a)** Diosmin and **b)** Hesperidin monitored during molecular dynamics simulations. Hydrogen bonds, hydrophobic, ionic and water bridges contacts are represented in green, violet, red and blue, respectively.

Figure S2. Plots of MM/GBSA trend for Diosmin (light blue line) and Hesperidin (salmon line) in complex to the proteasome chymotrypsin-like site, during 100 ns of MDs.

Figure S3. IC50 values of Hesperidin and Diosmin in MM cell lines. AMO wt and AMO BZB cells were treated with different concentrations of the two PCs for 48h, and cell viability assayed using the CTG method. IC50 values were calculated using GraphPad Prism 8 software and reported as mean of three independent experiments ±SD.

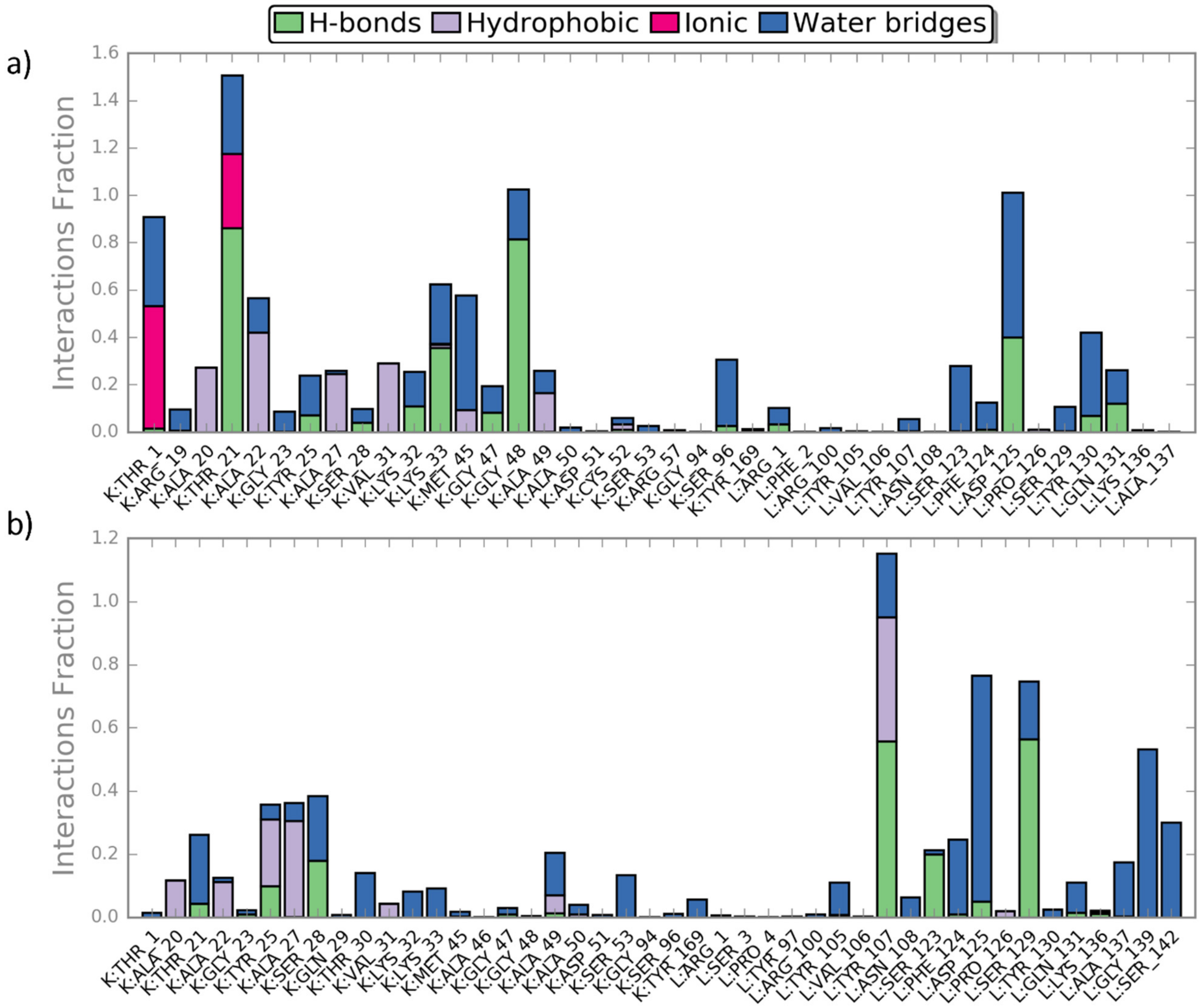

Figure S1. Protein-ligand interactions of **a)** Diosmin and **b)** Hesperidin monitored during molecular dynamics simulations. Hydrogen bonds, hydrophobic, ionic and water bridges contacts are represented in green, violet, red and blue, respectively.

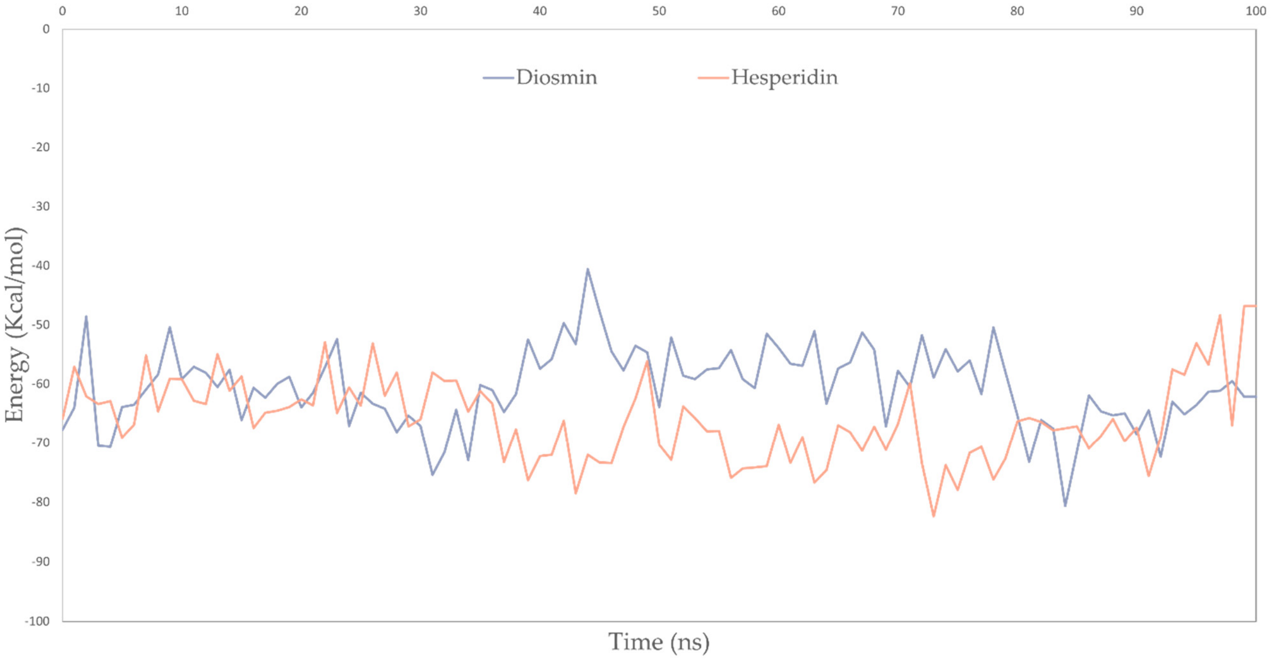

Figure S2. Plots of MM/GBSA trend for Diosmin (light blue line) and Hesperidin (salmon line) in complex to the proteasome chymotrypsin-like site, during 100 ns of MDs.

			**IC_{50}±SD**
**MM cell lines**	**AMO wt**	**Hesperidin**	150.9 μM±0.7
		**Diosmin**	80.34 μM±0.92
	**AMO BZB**	**Hesperidin**	531.2 μM±3.2
		**Diosmin**	437.3 μM±2.04

Figure S3. IC50 values of Hesperidin and Diosmin in MM cell lines. AMO wt and AMO BZB cells were treated with different concentrations of the two PCs for 48h, and cell viability assayed using the CTG method. IC50 values were calculated using GraphPad Prism 8 software and reported as mean of three independent experiments ±SD.
